# High-Frequency Notable HBV Mutations Identified in Blood Donors With Occult Hepatitis B Infection From Heyuan City of Southern China

**DOI:** 10.3389/fimmu.2022.754383

**Published:** 2022-05-13

**Authors:** Xianlin Ye, Lihua Liu, Lina Chen, Xianghui Nie, Lu Huang, Denghuang Ye, Jinfeng Zeng, Tong Li, Bin Li, Min Xu, Limin Chen

**Affiliations:** ^1^ Department of Laboratory, Shenzhen Blood Center, Shenzhen, China; ^2^ Department of Laboratory, Heyuan Blood Center, Heyuan, China; ^3^ The Joint Laboratory on Transfusion-Transmitted Diseases (TTDs) between Institute of Blood Transfusion, Chinese Academy of Medical Sciences and Nanning Blood Center, Nanning Blood Center, Nanning, China; ^4^ Provincial Key Laboratory for Transfusion-Transmitted Diseases, Institute of Blood Transfusion, Chinese Academy of Medical Sciences (CAMS) and Peking Union Medical College (PUMC), Chengdu, China

**Keywords:** occult hepatitis B virus infection (OBI), blood donors, gene mutations, mini pool (MP), nucleic acid testing (NAT)

## Abstract

**Background:**

All Chinese blood centers have implemented mini pool (MP) HBV nucleic acid testing (NAT) together with HBsAg ELISA in routine donor screening since 2015. The prevalence of occult hepatitis B virus infection (OBI) in donors from different regions varies, and the molecular characterization of the HBV DNA and clinical outcomes of these OBIs remain largely unexplored.

**Methods:**

Blood donations from Heyuan city in Southern China were screened by HBsAg ELISA and HBV MP8 NAT. Donations with HBsAg-/HBV DNA+ were collected for this study. Molecular characterizations of HBV DNAs were further analyzed by various DNA amplification assays including quantitative PCR (qPCR) and nested PCR, amplifying the basic core and pre-core promoter regions (BCP/PC). The HBsAg (S) region from HBV DNA was isolated by high-volume nucleic acid extraction. Notable mutations were identified by comparison to the HBV reference sequences. The clinical outcomes of the donors with OBIs were further followed for nearly 3 years.

**Results:**

Seventy OBIs from 44,592 donations (0.15%) that we identified and reported previously were enrolled for this current study. HBV sequences were obtained from 44/70 OBIs, and genotyping analysis showed that 42/44 (95.2%) OBIs were genotype B, and 2/44 (4.8%) were genotype C. Interestingly, mutation analysis revealed that various mutations including M133L/T, F134L, P142L, V168A, R169H, S174N, L175S, and V177A of HBV DNA affecting HBsAg detection were observed in genotype B OBIs. Two notable mutations, T47K and L53S, were identified in genotype C OBIs. Follow-up studies showed that 3/31 (9.7%) OBIs converted to HBsAg+ as chronic infections while 1/31 (3.2%) HBV DNA was undetectable (classified as recovery) and 27/31 (87.1%) remained as OBIs.

**Conclusion:**

Various notable mutations affecting HBsAg detection were observed in blood donors with OBIs in Heyuan city of Southern China. Follow-up studies showed that most OBIs remained as OBIs with fluctuating or low viral loads. Higher sensitive HBV ID NAT is recommended for donor screening to further reduce the transmission risk of OBIs.

## Introduction

Although transfusion-transmitted HBV infections have significantly reduced over the last decade due to the implementation of more appropriate screening approaches such as HBsAg ELISA combined with nucleic acid testing (NAT), occult hepatitis B virus infection (OBI) remains a major threat to blood safety. OBI is defined as the presence of very low levels of HBV DNA in plasma and/or in the liver, accompanied by undetectable hepatitis B surface antigen (HBsAg), with or without antibodies to hepatitis B core antigen (anti-HBc) or hepatitis B surface antibody (anti-HBs), outside the window period (WP) (1). OBI can be transmitted through blood transfusion, and in some cases, it can also progress to HBV infection when host immune function is declined or inhibited. This could contribute to chronic liver injury and the development of HBV-associated cirrhosis and hepatocellular carcinoma (2). Previous studies have indicated that the prevalence of OBI among Chinese volunteer blood donors is much higher than that of adjacent Asian countries and western countries, even by using ID NAT (3, 4), especially since MP NAT has been implemented nationwide in China since 2015 (5–8). A higher prevalence of OBIs in Chinese blood donors may pose a potential threat to blood safety.

Heyuan is a middle-sized city with about 3 million residents in the North-East part of Guangdong province, China. Since January 1, 2016, the Heyuan Blood Center has been implementing a nucleic acid test in MP8 format for HBV DNA, HCV RNA, and HIV RNA in parallel with two rounds of ELISA screening for HBsAg, anti-HCV, and anti-HIV in all donors. During the routine screening process, some donations with HBsAg-/HBV DNA+ were detected. Further clarification of these donations with inconsistent results in terms of the prevalence of OBIs, DNA mutations, and clinical outcomes is needed to ensure blood safety, especially in HBV high prevalent countries such as China.

In this current study, we performed HBV DNA mutation analysis on HBV DNA fragments amplified from HBsAg-/HBV DNA+ (OBI) donations. Various notable mutations that may affect HBsAg detection were identified. These mutations may affect HBsAg detection leading to OBIs that may potentially endanger blood safety. In addition, we performed a 3-year follow-up study to further analyze the clinical outcomes of these OBIs. Results from our follow-up study indicated that most OBIs remained as OBIs with fluctuating or low viral loads. Therefore, higher sensitive HBV ID NAT is recommended for donor screening to further reduce the transmission risk from donors with OBIs.

## Materials and Methods

### Blood Samples and Routine Screening

A total of 44,592 blood donations were collected by the Heyuan Blood Center between August 2017 and January 2019, and all the donors passed the physical examination, rapid test for HBsAg, ALT (Minray, Shenzhen), and hemoglobin, and dual ELISA assays [Bio-Rad (limit of detection, LOD: 0.5 IU/ml) and Chinese Livzon (LOD: 0.5 IU/ml) for HBsAg and anti-HIV; Beijing WanTai and Chinese Livzon for anti-HCV and TP] for routine screening as reported previously (9). All the informed consents were signed before blood sampling.

### NAT Screening

NAT screening was performed with MP8 pools as reported previously (9). Briefly, total nucleic acids from MP8 were extracted by ChiTas BSS1200 automatic machine followed by HBV DNA screening using a multiplex polymerase chain reaction kit (Haoyuan Biotech Co., Ltd. Shanghai; 1.2 ml, LOD: 10 IU/ml). All samples in reactive pools were retested individually (ID NAT), and any sample with an HBV-reactive result was defined as HBV DNA positive (HBsAg ELISA-/HBV DNA+).

### Molecular Characterization of HBV DNA

HBsAg-/HBV DNA+ samples were further analyzed by molecular characterization. Plasma (2.5 ml) was used for large volume viral nucleic acid extraction, and BCP/PC and S segments were amplified (for specific primers, please refer to **Table 1**) and sequenced as reported previously (10, 11). Virus loads were measured by qPCR (LOD, 5 IU/ml) (12). All the molecular characterization work was performed in a NAT research laboratory. International standards and strict institutional biosafety procedures were followed.

**Table 1:** PCR primers for HBV DNA used in this study.

Primer	Nucleotide sequences (5’-3’)	Position (nt)	Polarity
**BCP/PC**			
PC1	CATAAGAGGACTCTTGGACT	1,653–1,672	Forward
PC2	GAAAGAAGTCAGAAGGCAAA	1,954–1,973	Reverse
PC3	AATGTCAACGACCGACCTT	1,679–1,697	Forward
PC4	GAAAGAAGTCAGAAGGCAAA	1,954–1,973	Reverse
**S gene**			
PS1	CTCGTGTTACAGGCGGGGTTTTTC	191–214	Forward
PS2	CATCATCCATATAGCTGAAAGCCAAACA	721–748	Reverse
PS3	TTGTTGACAAGAATCCTCACAATACC	215–240	Forward
PS4	GCCCTACGAACCACTGAACAAATGG	686–710	Reverse

BCP/PC, basic core promoter/pre-core; S gene, HBV surface gene.

### OBI Confirmation

Two ELISA kits for HBsAg (Bio-Rad and Chinese Livzon) and ECLI for HBsAg (LOD, 0.05 IU/ml), anti-HBs (LOD, 2 IU/L), HBeAg, anti-HBe, and anti-HBc (Roche HBsAg II quant, Elecsys) were used for OBI confirmation. Donations with all negative results were defined as confirmatory negative. HBV NAT+ donations retested positive by at least any one of S, BCP/PC, and qPCR amplification were considered as HBV DNA confirmatory positive. Donations with HBsAg-/HBV DNA+ without any HBV sero-markers were regarded as WP infection, and those outside of WP were OBIs.

### HBV Genotyping and Identification of Notable Mutations

The DNA amplified products were sequenced by Shanghai Invitrogen Co., Ltd. (Guangzhou, China). MEGA7.1 program was performed for phylogenetic analysis and genotyping by using the neighbor-joining method with 1,000 bootstrap replications and Kimura 2-parameter mode. Notable mutations were calculated from the alignment of 124 genotype B and 95 genotype C reference sequences from HBsAg+ blood donors as previously reported (13). The ages of HBsAg+ blood donors used as genotype B and C reference sequences spanned from 18 to 58 years with an average age of 40.1 years and 19 to 59 years with an average age of 39.1 years, respectively.

### Statistical Analysis

Data were analyzed using statistics software STATA 16.0. Categorical variables and continuous variables were compared using Fisher’s exact test and the non-parametric Mann–Whitney test, respectively. Any *p*-value <0.05 was regarded as statistically significant.

## Results

### Confirmation of OBIs

**Figure 1:**
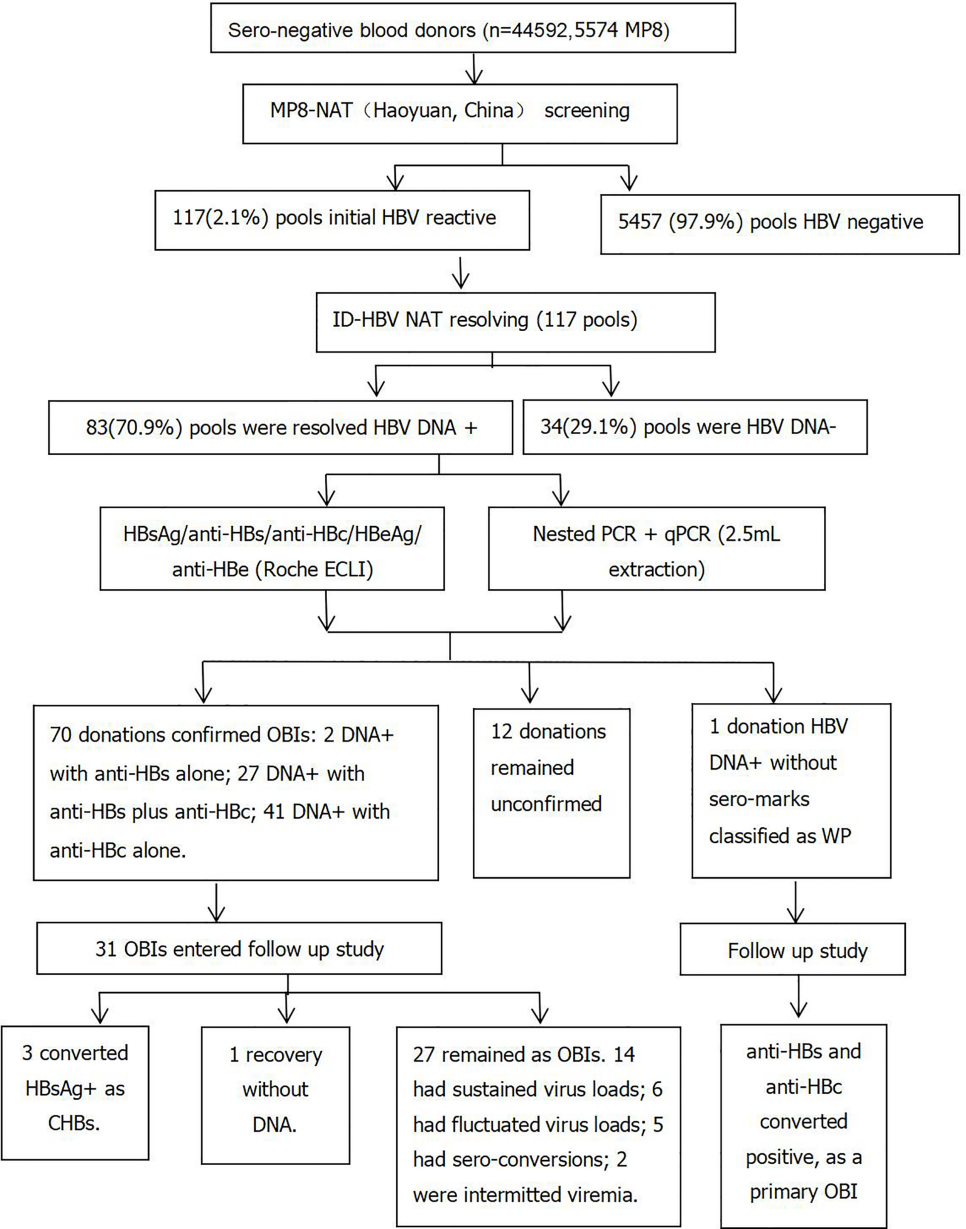
Flowchart of the study design.

Of 5,574 pools, 117 (2.1%) were identified as HBV DNA initial reactive, and ID NAT resolving was employed to further analyze these 117 MP NAT reactive pools. Of 117 pools, 83 (70.9%) were resolved HBV DNA+, and 34 pools (29.1%,112 donations) were HBV DNA- by ID HBV NAT as reported previously (9). In addition, one donation was confirmed WP, and 70 were-- confirmed as OBIs by a combination of various assays outlined in the *Materials and Methods*. The OBI yield rate was 0.16% (70/44,592, 1:637 donations), as shown in **Figure 1**.

### HBV Genotyping and Identification of Notable Mutations

HBV S sequences were successfully obtained from 43/70 confirmed OBIs, and genotyping analysis showed that 41/44 (95.2%) OBIs were genotype B, and 2/44 (4.8%) were genotype C. Furthermore, 1 WP donation was determined as genotype B. HBV S gene sequences including the major hydrophilic region (MHR) were compared with reference sequences (amino acids) statistically. No notable mutations were observed in 6 OBIs of genotype B from donors who came to Heyuan from other parts of China (immigrant donors) although 12 amino acid substitutions including two wild types were identified. Nevertheless, in 35 local genotype B OBIs, 11 mutations of M133L/S/T (20%), F134L/I (17.1%), P142L/S (5.7%), F158S (11.4%), K160N/R (5.7%), V168A/Q (28.6%), R169H/P (8.6%), F170S/V(5.7%), S174N/V(10.8%), L175H/S (10.8%), and V177A/Y (14.3%) were calculated as notable mutations (**Table 2**). Over 70% of these 11 notable mutations may affect the serological diagnosis of HBV, such as HBsAg detection, and 3 vaccine escape mutants, as well as 2 mutants causing failure in the production of HBV antibody, were also observed. In MHR, other mutations such as Q101R/K (8.6%), Q129H/R (11.4%), T131N/S (8.6%), M133L/S/T (22.9%), F134C/L/R/V (8.6%), T143M/S (11.4%), and Y161F/H (17.1%) were also identified in local OBIs. A total of 114 amino acid substitutions were present in 35 DNA sequences amplified from local OBIs, with the frequency of occurrence significantly higher (*p* < 0.05) than the references of genotype B (256 substitutions/124 strains) and the immigrant donors’ B strains (*p* = 0.000). Interestingly, both of the 2 genotype C sequences were from first-time immigrant donors who came from Henan and Hebei provinces in North China 5 years ago, harboring multiple mutations T47K, L53S, Q101R, I150T, and W165L, and L42P, T47K, L53S, L98R, S114T, I126M, P127S, M133T, S174N, and L175S, respectively, which included the notable mutations T47K (*p* = 0.006) and L53S (*p* = 0.041). There were no co-infections in all confirmed OBIs and WP.

**Table 2:** Notable mutations in the MHR and out of the MHR of genotype B in local donors with OBIs.

Position (aa)	Wild type	Occurrence			Affect serological diagnosis	Vaccine escape mutant	Failure in HBIg Therapy	References
		Background (*n* = 124)	Donors (*n* = 35)	*p*				
133	M	119 with 133M 1 with 133K 2 with 133L 2 with 133T	28 with 133M 4 with 133L 2 with 133T 1 with 133I	0.005	Yes (M133L, M133T)	Yes (M133L,M133T)	Yes (M133L)	(14, 15)
134	F	123 with 134 F 1 with 134L	29 with 134F 1 with 134C 2 with 134L 2 with 134R 1 with 134V	0.000	Yes (F134L)	ND	ND	(15)
142	P	124 with 142P	33 with 142P 1 with 142L 1 with 142S	0.047	Yes (P142L)	Yes (P142L/S)	Yes (P142L)	(16, 17)
158	F	123 with 158F 1 with 158L	31 with 158F 4 with 158S	0.009	ND	ND	ND	
160	K	124 with 160K	33 with 160K 1 with 160N 1 with 160R	0.047	ND	ND	ND	
168	V	122 with 168V 2 with 168I	25 with 168V 9 with 168A 1 with 168Q	0.00	Yes (V168A)	ND	ND	(18)
169	R	124 with 169R	32 with 169R 2 with 169H 1 with 169P	0.01	Yes (R169P)	ND	ND	(19)
170	F	124 with 170F	33 with 170F 1 with 170S 1 with 170V	0.047	ND	ND	ND	
174	S	122 with 174S 1 with 174N 1 with 174V	31 with 174S 1 with 174A 3 with 174N	0.02	Yes (S174N)	Yes(S174N)	ND	(16)
175	L	124 with 175 L	31 with 175 L 1 with 175H 3 with 175S	0.002	Yes (L175S)	ND	ND	(20)
177	V	123 with 177 V 1 with 177 A	30 with 177V 4 with 177A 1 with 177Y	0.002	Yes (V177A)	ND	ND	(21)

MHR, major hydrophilic region; OBI, occult hepatitis B infection; aa, amino acid; ND, not determined; HBIg, hepatitis B immunoglobulin.

### Follow-Up Study and Clinical Outcomes of OBIs

Thirty-one OBIs and 1 WP donor were followed up for nearly 3 years from November 2017 to October 2020. Three OBIs (9%, LH37, LH50, and LH68) converted to HBsAg+ confirmed by ECLI (0.07 IU/ml, 0.10 IU/ml, and 0.06 IU/ml, respectively) and ELISA within 1 year, and they were classified as chronic HBV infections. HBV DNA fell undetectable in 1 OBI (3.1%, LH20) with anti-HBs > 300 IU/ml, and this OBI was classified as recovery. The remaining 27 donors with OBIs remained as OBIs, among which 2 (6.3%, LH10 and LH36) had intermittent viremia during the follow-up period, and 6 (18.8%, LH31, LH35, LH47, LH55, LH66, and LH71) had 10-fold viral load fluctuations, while sero-marker conversion such as the presence of anti-HBs and anti-HBe occurred in 5 donors with OBI (16.1%, LH13, LH43, LH45, LH58, and LH53). Serological markers and viral loads of the remaining 14 donors (LH1, LH19, LH22, LH26, LH27, LH29, LH30, LH33, LH34, LH39, LH40, LH44, LH48, and LH65) remained stable (**Table 3**). The only WP (LH16) donor converted to anti-HBc+ with the virus load increasing to 97.32 IU/ml, and became an OBI. The range of anti-HBs and virus loads varies from 0 to >1,000 IU/L with a median of 15.73 IU/L and from 0 to 1207.14 IU/ml with a median of 25.34 IU/ml, respectively. Only 7/31 OBIs were identified as virus loads >100 IU/ml during the follow-up period, indicating that the viral loads in most OBI donors were relatively low, and this may pose difficulty in identifying them.

**Table 3:** Follow-up analysis of 31 OBIs and 1 WP donors.

Sample	F/R	Sero-markers					NAT	Virus load(IU/ml)	BCP/PC		S	Genotype
		HBsAg (IU/ml)	Anti-HBs(IU/L)	HBeAg	Anti-HBe	Anti-HBc						
LH1	1	<0.05	**-**	**-**	**-**	**+**	**+**	13.52		**+**	**+**	B
80d*		<0.05	**-**	**-**	**-**	**+**	**+**	57.32		**+**	**+**	
LH10	10	<0.05	>1,000	**-**	**+**	**+**	**+**	No Ct		**+**	**-**	
312d		<0.05	>1,000	**-**	**+**	**+**	**-**	No Ct		**-**	**-**	
551d		<0.05	>1,000	**-**	**+**	**+**	**+**	6.27		**-**	**-**	
LH13	16	<0.05	–	**-**	**+**	**+**	**+**	24.46		**-**	**+**	B
409d		<0.05	12.13	**-**	**+**	**+**	**+**	462.50		**-**	**+**	
575d		<0.05	13.38	**-**	**+**	**+**	**+**	34.64		**-**	**+**	
LH16	1	<0.05	–	**-**	**-**	**-**	**+**	26.43		**+**	**+**	B
322d		<0.05	361.30	**-**	**-**	**+**	**-**	97.32		**-**	**-**	
LH19	3	<0.05	47.85	**-**	**-**	**+**	**+**	No Ct		**+**	**+**	B
125d		<0.05	76.41	**-**	**-**	**+**	**+**	No Ct		**+**	**+**	
LH20	10	<0.05	289.9	**-**	**+**	**+**	**+**	6.79		**-**	**-**	
346d		<0.05	322.4	**-**	**+**	**+**	**-**	No Ct		**-**	**-**	
407d		<0.05	312.3	**-**	**+**	**+**	**-**	No Ct		**-**	**-**	
LH22	1	<0.05	252.7	**-**	**-**	**+**	**+**	15.16		–	**-**	
203d		<0.05	270.90	**-**	**-**	**+**	**+**	No Ct		–	**-**	
LH26	1	<0.05	44.14	**-**	**+**	**+**	**+**	4.13		**-**	**-**	
259d		<0.05	49.87	**-**	**+**	**+**	**-**	3.95		**+**	**-**	
287d		<0.05	56.55	**-**	**+**	**+**	**+**	16.45		**-**	**-**	
LH27	1	<0.05	–	**-**	**+**	**+**	**+**	12.34		–	**+**	B
95d		<0.05	–	**-**	**+**	**+**	**+**	1.95		–	**+**	
LH29	1	<0.05	15.68	**-**	**+**	**+**	**+**	9.88		**+**	**+**	B
242d		<0.05	35.38	**-**	**+**	**+**	**+**	4.36		**+**	**-**	
LH30	2	<0.05	>1,000	**-**	**+**	**+**	**+**	18.04		–	**+**	B
276d		<0.05	>1,000	**-**	**+**	**+**	**+**	41.79		–	**+**	
LH31	2	<0.05	–	**-**	**+**	**+**	**+**	33.75		**-**	**-**	B
140d		<0.05	–	**-**	**+**	**+**	**+**	580.36		**-**	**+**	
273d		<0.05	–	**-**	**+**	**+**	**+**	217.86		**-**	**+**	
LH33	1	<0.05	33.46	**-**	**+**	**+**	**+**	11.64		–	**+**	B
242d		<0.05	36.84	**-**	**+**	**+**	**+**	61.96		–	**+**	
447d		<0.05	40.51	**-**	**+**	**+**	**+**	7.84		–	**+**	
LH34	2	<0.05	–	**-**	**+**	**+**	**+**	426.79		**+**	**+**	B
346d		<0.05	–	**-**	**+**	**+**	**+**	1207.14		**+**	**+**	
LH35	4	<0.05	38.29	**-**	**+**	**+**	**+**	21.43		**-**	**+**	B
371d		<0.05	84.75	**-**	**+**	**+**	**+**	310.71		**-**	**+**	
508d		<0.05	54.44	**-**	**+**	**+**	**+**	13.00		**-**	**-**	
LH36	6	<0.05	>1,000	**-**	**-**	**+**	**+**	6.2		**-**	**-**	
170d		<0.05	>1,000	**-**	**-**	**+**	**-**	No Ct		**-**	**-**	
370d		<0.05	>1,000	**-**	**-**	**+**	**-**	5.45		**-**	**-**	
LH37	1	<0.05	–	**-**	**+**	**+**	**+**	410.71		**+**	**+**	B
332d		0.07	–	**-**	**+**	**+**	**+**	323.21		**-**	**-**	
LH39	1	<0.05	296.7	**-**	**+**	**+**	**+**	59.11		**-**	**+**	B
129d		<0.05	125.7	**-**	**+**	**+**	**+**	21.25		**-**	**-**	
LH40	1	<0.05	–	**-**	**-**	**+**	**+**	41.79		**+**	**-**	
214d		<0.05	–	**-**	**-**	**+**	**+**	No Ct		**-**	**-**	
LH43	10	<0.05	–	**-**	**+**	**+**	**+**	216.07		**+**	**+**	B
231d		<0.05	15.78	**-**	**+**	**+**	**+**	139.11		**+**	**+**	
LH44	3	<0.05	12.19	**-**	**-**	**+**	**+**	38.39		**-**	**+**	B
120d		<0.05	17.86	**-**	**-**	**+**	**+**	5.55		**-**	**-**	
LH45	1	<0.05	–	**-**	**-**	**+**	**+**	No Ct		**+**	**+**	B
308d		<0.05	19.12	**-**	**-**	**+**	**+**	11.21		**+**	**+**	
LH47	2	<0.05	47.56	**-**	**-**	**+**	**+**	4.23		**-**	**-**	B
248d		<0.05	160.30	**-**	**-**	**+**	**+**	326.79		**-**	**+**	
LH48	3	<0.05	–	**-**	**+**	**+**	**+**	15.21		**-**	**+**	B
207d		<0.05	–	**-**	**+**	**+**	**+**	No Ct		**-**	**+**	
246d		<0.05	–	**-**	**+**	**+**	**+**	12.79		**-**	**-**	
LH50	1	<0.05	–	**-**	**+**	**+**	**+**	550.00		**-**	**+**	B
311d		0.10	–	**-**	**+**	**+**	**+**	161.96		**-**	**+**	
LH53	5	<0.05	59.28	**-**	**-**	**+**	**+**	207.14		**+**	**+**	B
484d		<0.05	53.57	**-**	**+**	**+**	**-**	49.29		**+**	**-**	
LH55	1	<0.05	–	**-**	**+**	**+**	**+**	273.21		**-**	**-**	
868d		<0.05	–	**-**	**+**	**+**	**+**	3.88		**-**	**-**	
LH58	3	<0.05	–	**-**	**-**	**+**	**+**	237.50		**+**	**+**	B
507d		<0.05	–	**-**	**-**	**+**	**+**	185.71		**+**	**+**	
708d		<0.05	14.08	**-**	**-**	**+**	**+**	3.80		**+**	**+**	
LH65	1	<0.05	–	**-**	**+**	**+**	**+**	182.14		**+**	**+**	B
244d		<0.05	–	**-**	**+**	**+**	**+**	150.00		**+**	**+**	
LH66	1	<0.05	20.46	**-**	**-**	**+**	**+**	178.39		**-**	**-**	
317d		<0.05	44.61	**-**	**-**	**+**	**+**	No Ct		**-**	**-**	
LH68	1	<0.05	**-**	**-**	**+**	**+**	**+**	208.93		**-**	**-**	
257d		0.06	**-**	**-**	**+**	**+**	**+**	23.57		**-**	**-**	
LH71	3	<0.05	–	**-**	**+**	**+**	**+**	453.57		**+**	**+**	B
135d		<0.05	–	**-**	**+**	**+**	**+**	23.75		**+**	**+**	

OBI, occult hepatitis B infection; WP, window period; F/R, First time donor/Repeat donor; NAT, Nucleic acid test; BCP/PC, basic core promoter/pre-core; anti-HBc, hepatitis B core antibody; anti-HBs, hepatitis B surface antibody; anti-HBe, hepatitis B e antibody; No Ct, HBV DNA undetected by qPCR; d*, no. of days post index; NAT, Haoyuan ID NAT; BCP/PC, nested PCR for BCP/PC; S, nested PCR for S. anti-HBs < 10 IU/L was regarded as negative, and HBsAg < 0.05 IU/ml was regarded as negative.

## Discussion

Chronic hepatitis B virus (HBV) infection remains a major public health problem, particularly in China. Since the introduction of the universal HBV vaccination program, by the Chinese government over two decades ago, there has been a significant reduction in the prevalence of HBsAg from 10% to <1% in children (21). However, the prevalence of HBsAg in native residents in Guangdong province, where Heyuan is located, was as high as 11.1% in 2006 (22) and 8.76% in 2015 in the general population (23), posing safety concerns for blood donation. Therefore, stricter donor screening is essential to ensure blood safety.

In countries where HBV infection is prevalent, appropriate screening strategies are needed. Although ID NAT is capable of detecting the low virus loads of HBV DNA with high automation, MP NATs are still used more often in China due to their lower cost. Donors with OBI detected by either ID or MP NAT pose a potential threat to blood safety. A Chinese multicenter study performed on 826,044 serologic-negative donations in MPs of six indicated that 0.07% of donors were classified as OBIs (24). In line with this result, 0.08% of donors were identified as OBIs by ID NAT screening in 10 Chinese blood centers (25). The prevalence of OBI in blood donors varied considerably depending on many factors, such as HBV prevalence in different regions, the proportion of the repeated or the first-time donors, NAT sensitivity, and the pooling strategy used. For example, the prevalence of OBIs in blood donors was 0.026% in Shenzhen (5), 0.029% in Hong Kong (3), 0.0078% in Spain (26), 0.004% in Slovenia (26), and 0.00067% in Germany (26), whereas we identified that 0.15% of donors were OBIs in the Heyuan Blood Center using MP8 NAT and various molecular and serological assays (9). This prevalence is nearly twice as high as that in other regions of China (24, 25). One main reason for the higher prevalence of OBIs in Heyuan was probably that the HBsAg prevalence in Heyuan blood donors of 1.15% was higher than the national average of 0.7% (27). The prevalence of OBI in blood donors from Heyuan city is also significantly higher than that in Qingdao (0.06%, MP6, *p* < 0.05) (28), Shenzhen (0.026%, MP8, *p* < 0.05) (5), Hong Kong (0.029%, ID NAT) (3), Pakistan (0.028%, MP6) (29), Thailand (0.024%, MP6) (7), USA (0.00018%) (30), and European countries having a low prevalence of HBV (0.008%) (31).

Our current study focuses on the mutation analysis of HBV DNA amplified from donors with confirmed OBIs and a relatively long period of follow up study to look at the clinical outcomes of these OBIs. Data from our study demonstrated that a high frequency of OBIs was detected in the blood donors in Heyuan city of Southern China, and various notable mutations affecting HBsAg detection were identified.

Although anti-HBs can neutralize HBV infectivity and clear circulating HBsAg and infectious HBV particles from peripheral blood (32), HBV DNA in the presence of anti-HBs may be infectious. Though only two documented transfusion-transmitted HBV infections have been reported from donations with anti-HBs, the titer of anti-HBs is critical to determine whether these donations are safe to donate or not. The titer of anti-HBs in the above 2 infectious donations was relatively low, and the higher anti-HBs was only 29.6 IU/L (33, 34). Accordingly, some countries such as Australia and Canada proposed that blood donation with anti-HBs levels higher than 100 IU/L (>200 IU/L in Japan) be considered safe (35). Nevertheless, data from organ transplantation also clearly demonstrated that HBV DNA in the presence of anti-HBs could be infectious in immunosuppressed patients (36). In the current study and our previous studies, only 9/70 (12.9%) OBIs were observed with anti-HBs >100 IU/L. More importantly, 40/70 (57.1%) OBIs were anti-HBc positive alone, interpreted as more infectious than those with low levels of anti-HBs, and these data may signify a higher threat to blood safety in Heyuan city (9). In addition, for 70 confirmed OBIs from 117 initial HBV DNA reactive pools, only 27 can be detected by three alternative NAT assays, and the median of virus load (25.71 IU/ml) was far below the LOD of the MP8 ID NAT (66.7 IU/per sample) (9), reflecting that most of the OBIs were detected by the probability determined by Poisson distribution (37). This implies that a number of OBIs with extremely low viral loads could not be intercepted by the HBV MP8 NAT. During the follow-up period, most OBIs showed low virus loads, intermittent viremia, or undetectable virus by NATs, suggesting that a certain proportion of OBIs were missed due to insufficient assay sensitivity and/or MP format, which is supported by another study performed in China (13). Additionally, an American comprehensive study from 22.4 million blood donors revealed that only 43/404 (10.6%) OBIs could be detected by MP NAT, and most OBIs (361/404, 89.4%) could only be identified by ID NAT (30), raising an urgent need for more optimized screening strategies and higher sensitive HBV ID NAT in high HBV prevalent regions.

The clinical outcomes of the donors with OBI and their infectivity can be followed to further understand the natural transition of OBIs. We successfully followed 31 OBIs and one donor in WP. Three cases (9.7%) were shown to have sero-conversion to HBsAg+, and therefore progressed to chronic hepatitis B infections (CHBs). A low level of HBsAg might indicate that some OBIs could be chronic HBV carriers when HBsAg is no longer detectable, but HBV DNA may still be present with a very low viral load (10). The two cases we followed had intermittent viremia with high levels of anti-HBs, suggesting that these cases might occur largely in individuals who have recovered from HBV infection but are unable to mount an effective immune response to eliminate the virus (38). HBV DNA from one donor in our follow-up with anti-HBs, dropped to undetectable levels without sero-conversion, which might be indicative of recovery after HBV infection because anti-HBs increased up to more than 300 IU/ml (39). Sero-conversions were observed in 5 cases, reflecting resolution from acute infections.

HBV DNA integration not only interferes with the cell cycle of hepatocytes and/or results in the production of pro-oncogenic proteins, such as HBx and mutated surface proteins that can lead to the development of hepatocellular carcinoma (HCC), but also can cause low-grade hepatic necroinflammation contributing to liver fibrosis and cirrhosis (40). Therefore, a longer follow-up for the outcomes of OBIs is essential for understanding proper management.

In this study, 41/43 (95.3%) OBIs were genotype B, which is similar to our previous study (14/15,93.3%) (41), and the remaining two immigrants were from Northern China. This result is consistent with a nationwide survey reporting that HBV genotype B was predominant in Southern China, while genotype C was the major genotype in Northern China (42). HBV genotype is an important risk factor for developing HCC in HBV-infected individuals (43). One study from Taiwan following over 3,000 HBV-infected individuals with either genotype B1 or C reported that while HCC incidence was significantly higher in those with genotype C, the incidence of HCC was high in individuals over 40 years with genotype B1 (44). Interestingly, genotype B may be associated with a more benign clinical course than genotype C or D, because genotype B responds to IFN-based therapies better although the response rate to nucleoside analogue therapy does not appear to be influenced by HBV genotypes (45).

HBV S gene mutations reduce HBsAg antigenicity and/or elicit low-affinity or non-neutralizing antibodies that may interfere with HBsAg detection by routine ELISA; this false negative in HBsAg detection often leads to OBI (46). As such, a more detailed analysis of S gene mutations from OBI donations is important. In this context, various notable mutations affecting HBsAg detection such as M133L/T, F134L, P142L, V168A, R169H, S174N, L175S, and V177A were observed in the local genotype B blood donors with OBI. Convincingly, some notable mutations such as V168A, R169P, S174N, L175S, and V177A were also observed in blood donors from our previous study (13), and this detection evasion strategy employed by the virus might be one of the main reasons for the relatively higher OBI yield rate in Heyuan.

In conclusion, we identified by DNA sequencing a large number of notable mutations affecting HBsAg detection in local blood donors with OBI of genotype B. A follow-up study showed that 9.7% of OBIs had progressed to CHBs while most OBIs remained as OBIs with fluctuating or low viral loads. Although testing of other surrogate markers and detailed donor questionnaires regarding the risk factors for HBV infection are useful, more sensitive HBV ID NAT is strongly recommended for donor screening to reduce the transmission risk of OBIs in HBV high endemic regions.

## Data Availability Statement

The original contributions presented in the study are included in the article/supplementary material. Further inquiries can be directed to the corresponding authors.

## Ethics Statement

This study was approved by the Ethics Committee of Shenzhen Blood Center. All methods were carried out in accordance with relevant guidelines and regulations.

## Author Contributions

XY and LL designed the experiments and wrote and reviewed the manuscript. LimC, BL, and MX reviewed, revised, and edited the manuscript. LinC, BL, XN, LH, DY, TL, and JZ participated in the study design, performed the experiments, and collected and analyzed the data. All authors contributed to the article and approved the submitted version.

## Funding

This work was supported by the Nature and Science Fund of Shenzhen and Guangdong (JCYJ20190806112201646 and 2021A1515010979) and Shenzhen Key Medical Discipline Construction Fund (SZXK070) to XY and CAMS Initiative for Innovative Medicine (CAMS-2016-I2M-3-025 and CAMS-2017-I2M-B&R-15), the National Key Research and Development Program (2018YFE0107500), and Science and Technology Partnership Program, Ministry of Science and Technology of China (KY201904011) to LC.

## Conflict of Interest

The authors declare that the research was conducted in the absence of any commercial or financial relationships that could be construed as a potential conflict of interest.

## Publisher’s Note

All claims expressed in this article are solely those of the authors and do not necessarily represent those of their affiliated organizations, or those of the publisher, the editors and the reviewers. Any product that may be evaluated in this article, or claim that may be made by its manufacturer, is not guaranteed or endorsed by the publisher.

## Abbreviations

HBV, hepatitis B virus; HBsAg, hepatitis B surface antigen; anti-HBc, hepatitis B core antibody; anti-HBs, hepatitis B surface antibody; OBI, occult hepatitis B infection; CHB, chronic hepatitis B; NAT, nucleic acid testing; ELISA(s), enzyme-linked immuno-absorbent assay(s); ECLI, electrochemiluminescence immunoassay; ID, individual donation; MP, mini pool; PCR, polymerase chain reaction; LOD, limit of detection; S/CO, sample to cutoff; BCP/PC, basic core promoter/pre-core; MHR, major hydrophilic region.
